# Prevalence and acquisition of MRSA amongst patients admitted to a tertiary-care hospital in brazil

**DOI:** 10.1186/1471-2334-10-328

**Published:** 2010-11-14

**Authors:** Helena B Santos, Denise P Machado, Suzi A Camey, Ricardo S Kuchenbecker, Afonso L Barth, Mário B Wagner

**Affiliations:** 1 Hospital de Clínicas de Porto Alegre, Porto Alegre, Brazil; 2 Pós-Graduação em Epidemiologia, Universidade Federal do Rio Grande do Sul, Porto Alegre, Brazil; 3 Instituto de Matemática- Universidade Federal do Rio Grande do Sul, Porto Alegre, Brazil; 4 Faculdade de Farmácia, Universidade Federal do Rio Grande do Sul, Porto Alegre, Brazil

## Abstract

**Background:**

There are few studies in Brazil that address baseline prevalence of MRSA colonization and associated risk factors at hospital admission, or the incidence of nosocomial colonization. We report a prospective study in a tertiary-care, university-affiliated hospital to implement a new MRSA control policy at the institution.

**Methods:**

A cohort of randomly selected patients admitted to emergency and clinical wards at our hospital was followed until discharge. Nasal swabs were taken for identification of MRSA-colonized patients and detection of SCC*mecA *in positive cultures, at admission and weekly thereafter. Multivariate analysis using a log-binomial analysis was used to identify risk factors for colonization.

**Results:**

After screening 297 adult patients and 176 pediatric patients, the prevalence of MRSA at admission was 6.1% (95%CI, 3.6% to 9.4%), in the adult population and 2.3% (95%CI, 0.6% to 5.7%), for children. From multivariate analysis, the risk factors associated with colonization in adults were: age above 60 years (*P *= 0.019) and hospitalization in the previous year (*P *= 0.022). Incidence analysis was performed in 276 MRSA-negative patients (175 adults and 101 children). Acquisition rate was 5.5/1,000 patient-days for adults (95%CI, 3.4 to 8.5/1,000 patients-days), and 1.1/1,000 patient-days for children (95%CI, 0.1 to 4.0/1,000 patients-days).

**Conclusions:**

The identification of MRSA carriers is a step towards establishing a control policy for MRSA, and helps to identify measures needed to reduce colonization pressure and to decrease the high acquisition rate in hospitalized patients.

## Background

Methicillin-resistant *Staphylococcus aureus *(MRSA) is cross-transmitted in hospital settings, and has a high impact not only on patient morbidity and mortality but also on hospitalization costs. Worldwide, it has been endemic in many healthcare facilities since the 1990 s [1]. MRSA remains a major pathogen in nosocomial infections in developing countries [2] and in Latin America, according to SENTRY [3].

The fact that a patient can harbor MRSA at hospital admission has consequences not just for the choice of patient treatment: it also impacts on the effectiveness of infection control in the hospital. MRSA reservoir at hospital can make other measures of infection control not as effective, thereby causing pathogen transmission to continue [4]. Guidelines aimed at controlling the spread of MRSA therefore propose to systematically search for colonized patients, and then to isolate and decolonize them [5]. This policy has not been tested rigorously in methodologically-sound randomized trials, and most of the evidence comes from observational and quasi-experimental studies [6]. There is also concern about the cost of such measures and the lack of available rooms for isolation [7].

Recently-published studies have arrived at different results regarding identification and isolation of MRSA colonized patients. These differences may be attributable to many factors, ranging from differences between the settings and patients to methodological issues, and the multifaceted nature of infection control practices. In one study, universal MRSA surveillance reduced the infection risk during hospitalization and 30 days after discharge [8], while another study evaluated MRSA screening at admission in surgical patients in an endemic setting of MRSA, and found no decrease in surgical site infections and nosocomial acquisition of MRSA [9]. Yet another study in an intensive care unit (ICU) compared two interventions to reduce transmission of MRSA, after identification of colonized patients by the pathogen: cohort-isolation o single room isolation, and found no difference in cross-transmission between the two periods [10].

In Brazil, MRSA is endemic in hospitals [3], but few studies have looked at MRSA colonization. One study that screened patients in an emergency department found 0.7% of patients colonized with MRSA at admission [11]. In ICUs, one study reported 13% of patients colonized at admission [12], and another [13] found 46% patients colonized. In this ICU, 51% of non-colonized patients acquired MRSA colonization [13].

We agree that the identification of baseline rates and the associated risk factors of colonization are necessary to estimate the burden of colonization and the demands for isolation facilities [14], and to help adopt a cost-effective strategy of patient screening which would take into consideration the population prevalence of MRSA and the structure of the hospital [15]. Thus, use of own data, obtained from a study that uses local resources can help to propose a policy that fits the institutional needs and lead to satisfactory outcomes regarding MRSA control.

The present study aims to estimate the prevalence of MRSA colonization and infection in clinical adult and pediatric patients at the time of admission to hospital; the incidence of colonization and infection during hospitalization; and the potential risk factors for both, in a hospital in southern Brazil, in order to obtain information to support infection control planning for MRSA policy in the institution.

## Methods

### Study Setting and Population

A prospective cohort study was made of patients admitted to the Hospital de Clínicas in Porto Alegre, an urban tertiary-care, public university-affiliated teaching hospital, with 749 beds, with three adult ICUs, one pediatric and one neonatal ICU. The hospital provides care in medical and surgical specialties. The infection control team performs hospital-wide, hospital-acquired infection (HAI) surveillance in all wards and in the hospital ICUs. There is a protocol for vancomycin-resistant enterococci (VRE) screening in patients transferred from other healthcare facilities, but there is no screening for MRSA colonization. Patients with clinical cultures who grew resistant micro-organisms were subject to isolation precautions and were placed in isolation rooms, depending on the availability of the latter.

We randomly selected adult (patients above 14 years old) and pediatric (14 years old or less) patients, in their first 72 hours of admission to the hospital. For logistic reasons, patients were selected from Mondays to Thursdays, since information collected for the hospital's corporate database indicates that there was no difference in clinical characteristics between patients admitted to the hospital on weekdays, and those admitted at weekends. On each of these days, one of the researchers (HBS) checked the admission of clinical patients who had arrived on the previous day. Surgical, gynecology/obstetric, neonatology or psychiatric patients were not included. For patients admitted to the emergency room (ER), the researcher asked the assistant physician to identify which of those were expected to stay for longer than 48 h in the hospital, so as to include them in the list. A number was assigned to each patient in two separated lists, one for the adult patients and another one for pediatric patients. Three adults and two children were randomly selected from respective list each day. Informed consent was obtained from patients or their caregivers. If a patient could not sign the informed consent and we were unable to contact a relative, we substituted that person for another randomly-selected patient from the list, whenever possible. Patients who had been selected in a previous admission could have been included, but were excluded from the statistical analysis.

### Data collection

After selection, and after informed consent had been given, trained researchers interviewed patients or those responsible for them. A standard form was used to record data. The researchers obtained clinical information from assistant physicians and medical residents, and from electronic medical records about diagnosis to calculate a Charlson Comorbidity Index. Information regarding previous use of drugs and previous hospitalizations were obtained from patients or the persons responsible for them. We chose not to obtain this information from electronic medical records because not all patients had previous hospitalization at the hospital, use of hospital records could therefore have led to a measurement bias.

A sample from the anterior nare was collected to identify MRSA colonization, with a swab moistened in sterile saline solution. Patients were followed until hospital discharge, with swabs collected weekly until eight samples had been collected and, whenever possible, another was collected at the moment of discharge. Swab results were kept undisclosed until the end of the study. The Charlson Comorbidity Score (CCS) was used for risk adjustment [16].

To obtain information about MRSA infection at admission or during hospitalization, we also searched for MRSA in clinical cultures collected as part of the routine hospital care provided to the selected patients.

### Microbiologic Methods

The swabs were transported in a Stuart's medium to the microbiology laboratory. All samples were processed using a mannitol salt agar medium (Himedia) with 6 ug/mL of oxacillin. The plates of mannitol salt agar were incubated at 35°C for 24 to 48 hours. Colonies with indication of mannitol fermentation were submitted to the coagulase test to confirm *S. aureus *identification. These colonies were also tested with a 30 μg cefoxitin disc (Oxoid) in a Mueller Hinton plate for oxacillin resistance [17].

For PCR detection of SCC*mecA*, the technique described by Vannuffel et al. [18] was used.

### Definitions

A patient was deemed to be colonized when a screening specimen grew MRSA; and was deemed infected when a clinical specimen was positive for MRSA. Admission cultures were those taken up to 72 hours after hospital admission. In Brazil, the Brazilian National Health Regulatory Agency (ANVISA) uses this cutoff as a criterion for nosocomial infection [19].

### Statistical Analysis

Sample size was calculated with EpiInfo 6.0, considering a prevalence of 5% of colonized patients at admission [14,20,21] and confidence level of 95%. The study sample was stratified into two groups, adult and pediatric, and we calculated a sample size of 300 adults and 200 pediatric patients. When data for the entire sample were shown, the results of the strata were weighted according to the percentage of clinical adult and pediatric patients in hospital.

Analyses were performed using SPSS 15.0 for Windows (SPSS Inc.). We used chi-square tests, with Fisher's Exact Test if indicated, for categorical variables, and *Student's t-test *or the *Mann-Whitney U test *for continuous variables in univariate analyses. To determine potential risk factors for colonization at admission or acquisition of colonization, we performed, respectively, log-binomial regression [22] and survival analysis with Cox regression. The multivariate analysis included variables with *P *< 0.20 in the univariate analysis, and the final model included those with *P < 0,05*. The study was submitted and was approved by the hospital's Ethics Committee (project number 05-341), and was approved.

## Results

**Figure 1:**
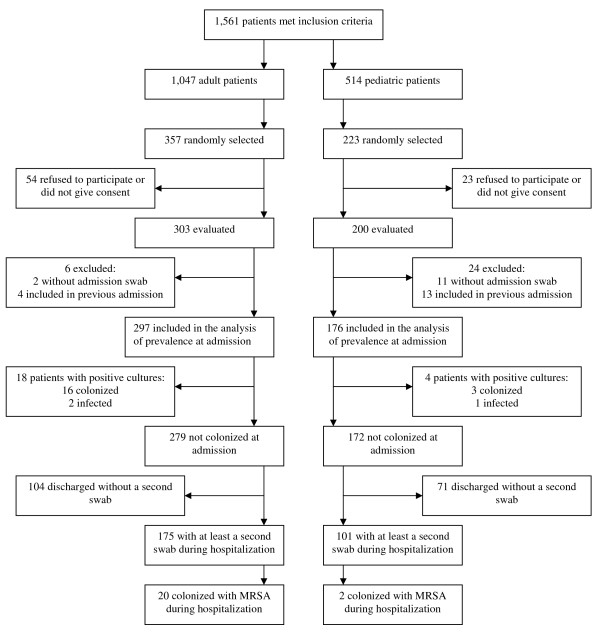
**Patients' flow chart in the study**.

From May, 2006 to March, 2007, 1,561 patients met the inclusion criteria, and 580 were selected (Figure 1). The final sample consisted of 473 patients (297 adults, 176 children). For incidence analysis, we followed 276 patients who had at least a second swab during hospitalization, and who had a negative culture at admission. There were 5,414 days of hospitalization. The first swabs were collected within 24 hours of admission for 377 patients, up 48 hours after admission for 95 patients and up 72 hours of admission for one patient.

Sixteen adult patients had been colonized with MRSA at admission (5.4%; 95%CI, 3.1% to 8.6%), and two were infected as shown by blood and skin ulcer secretion. Three (1.7%; 95%CI 0.4% to 4.9%) pediatric patients were colonized and one was infected (sputum) at admission. Weighted prevalence of MRSA at admission for the whole sample was 5.3% (95%CI, 4.5 to 6.2%). None of the patients with clinical cultures positive for MRSA at admission had positive nasal swabs, and none of the colonized patients developed a clinical infection with MRSA while hospitalized, although one of them received empirical vancomycin.

Table 1 shows the characteristics of the patients at admission, according to their MRSA status (positive or negative). For the children, none of the characteristics was associated with colonization or infection with MRSA. Hospitalization in the last year (PR = 5.3, 95%CI 1.2 to 22.5, *P *= 0.011) and age above 60 years (PR = 2.8, 95%CI 1.1 to 7.2, *P *= 0.046) were factors associated with colonization in adults in the univariate analysis.

**Table 1:** Characteristics of admitted patients included in the study

Variable	Pediatric patients		Adult patients	
	Negative	Positive	Negative	Positive
	(N = 172)	(N = 4)	(N = 279)	(N = 18)
Male sex (%)	101 (58.7)	2 (50.0)	157(56.3)	10 (55.6)
Mean age, years (SD)	4.1 (4.2)	7.8 (5.1)	54.1 (18.3)	59.8 (22.7)
Transfer (%)#	18 (10.5)	0 (0.0)	12 (4.3)	2 (11.1)
Admission by ER (%)	105 (61.0)	2 (50.0)	223 (79.9)	15 (83.3)
Median LOS, days (IQR)	8.0 (12.0)	11.0 (32.0)	11.0 (16.0)	14.0 (12.0)
Hospital admission last year (%) Median N admissions(IQR)*	116 (67.4) 1.0 (3.0)	4 (100.0) 2.0 (5.0)	163 (58.4) 1 (2.0)	16 (88.9) 4(5.0)
Ambulatory care (%)§	27 (15.7)	1 (25.0)	50 (17.9)	4 (22.2)
Previous MRSA (%)	30 (17.4)	2 (50.0)	22 (7.9)	1 (5.6)
Antimicrobials 2 wks before (%)	66 (38.4)	2 (50.0)	75 (26.9)	9 (50.0)
Corticosteroids 2 wks before (%)	30 (17.4)	1(25.0)	23 (8.2)	3 (16.7)
Immunosupressors 2 wks before (%)	4 (2.3)	0 (0.0)	15 (5.4)	0 (0.0)
Skin lesions (%)	38 (22.1)	1 (25.0)	55 (19.7)	3 (16.7)
HIV infection (%)	5 (2.9)	0 (0)	31 (11.1)	1 (5.6)
Diabetes mellitus (%)	2 (1.2)	1 (25.0)	53 (19.0)	4 (22.2)
Any neoplasia (%)	33 (19.2)	1 (25.0)	4 (30.1)	5 (27.8)
Pulmonary disease (%)	37 (21.6)	2 (50.0)	31 (11.1)	1 (5.6)

ER: Emergency Room, LOS: length of stay, IQR: interquartile range, wks: weeks, MRSA: Methicillin-resistant *Staphylococcus aureus *HIV: human immunodeficiency virus

# Transfer from another hospital or long term care facility

* in patients with at least one admission in the last year

§ Ambulatory care due chronic condition: dialysis and/or chemotherapy and/or radiotherapy

¶ Cystic Fibrosis, Asthma or Chronic Obstructive Pulmonary Disease

Multivariate analysis with log-binomial regression for the adults included the variables age above 60 years, previous admission, previous use of antibiotics and transfer from another hospital or from long term care facility (Table 2); both age above 60 years and previous hospital admission were associated with an increased risk of MRSA colonization at admission (PR = 3.0, 95%CI 1.2 to 7.7 and PR = 5.6, 95%CI 1.3 to 23.9, respectively).

**Table 2:** Multivariate analysis with risk factors for prevalence of MRSA colonization at admission in adults - log-binomial regression (N = 297)

Variable	PR (95%CI)
Age above 60 years	3.0 (1.2-7.7)
Transfer from another hospital or long term care facility	2.9 (0.8-11.0)
Use of antimicrobials 2 wks before	2.3 (0.9-5.5)
Hospital admission last year	5.6 (1.3-23.9)

MRSA: Methicillin-resistant *Staphylococcus aureus*

Weighted incidence of MRSA was 9.6% (95%CI 8.1 to 11.1) for the patients followed during hospitalization. Twenty adults acquired colonization during hospitalization (incidence rate, 5.5/1,000 patient-days, 95%CI, 3.4 to 8.5), and two children became colonized (incidence rate, 1.1/1,000 patient-days (95%CI, 0.1 to 4.0).

The incidence of colonization increased with increase length of stay, and was statistically significant (*P *< 0.001). It reached 2.9% in week two, 4.9% in week three, 2.4% in week four, 8.9% in week five, zero in week six, 9.1% in week seven and zero in week eight. For those patients with negative cultures at admission and have acquired colonization, the median number of days from admission until positive cultures were obtained from children was 8 days, and 15 days for adults. Adult patients with positive screening cultures had spent a mean of 4.05 days (median, 3) in the emergency, while patients with negative cultures, 2.72 days (median, 2), (*P *= 0.147 for the comparison, using a Mann-Whitney test).

The crude survival analysis with adult patients could not identify any risk factors associated with acquisition of colonization or infection; a multivariate analysis included Charlson Comorbidity Score (HR 1.1, 95%CI 0.7 to 1.6), presence of HIV infection (HR 1.2, 95%CI 0.1 to 10.1) and admission by ER (HR 3.0, 95%CI 0.7 to 13.3). None of these factors was associated with acquisition of MRSA (Table 3).

**Table 3:** Multivariate analysis with risk factors for acquisition of MRSA colonization during hospitalization in adults - Cox regression (N = 175)

Variable	HR (95%CI)
Admission by ER	2.9 (0.6-12.7)
HIV infection	1.1 (0.1-9.5)
Ward transfers	1.3 (0.7-2.4)
CCI	1.1 (0.7-1.6)

MRSA: Methicillin-resistant *Staphylococcus aureus*: ER emergency room; HIV: human immunodeficiency virus; CCI: Charlson Comorbidity Index

We identified the genotype of 36 of the 41 patients with positive swabs. For the positive patients at admission, the SCC*mecA *genes present were type I in five patients and type III in eight. For the patients that acquired colonization, there were SCC*mecA *type I in seven patients, SCC*mecA *type III in twelve and SCC*mecA *type IV in two.

## Discussion

Our study found a prevalence of MRSA colonization at admission that is higher than that previous reported in patients admitted to emergency service in Brazil [11], and may reflect the chronic nature of patients in a tertiary care hospital. Sixty-four percent of our sample had had at least one hospitalization in the previous year, and this was associated with colonization at admission. The results of SCC*mecA*, which mainly disclosed those types associated with hospital strains, also confirmed this profile. Moreover, the two strains with SCC*mecA *type IV acquired after admission are in agreement with a study that reported hospital dissemination of CA-MRSA in hospitals in Detroit [23].

However, even thought 70% of all pediatric patients had been admitted to a hospital in the last year, 19% of them with a neoplastic disease, the prevalence of colonization proved to be low in children. In Brazil, another study also reported low prevalence of MRSA in hospitalized children younger than five years (1%), and all strains harbored SCC*mecA *type III [24]. The number of positive children was too small for performing a multivariate analysis.

The two risk factors found for colonization at admission in our adult patients have been reported elsewhere in the literature [25]. However, there are also other risk factors associated with MRSA colonization that have been reported, but we found no association with HIV infection or with treatment for hemodyalisis. This may be due to the small number of those patients in the population under study, and the consequent lack of power to identify such associations, which is a limitation of our study. Another limitation may be related to the lack of association between previous drug use (i.e. antibiotic, corticosteroids or other immunosupressors) and MRSA harboring. This may reflect an information bias because most patients in the study were not able to report the name or type of the drugs that they had been using in the recent past.

The high incidence of colonization between hospitalized patients is a matter of concern. Two other studies in Brazil have also shown a high incidence rate of colonization [12,13], although they were in ICU settings, where patients are more exposed to invasive procedures (intravenous catheters, urinary catheters and mechanical ventilation) with higher rates of colonization. We could not identify any particular risk factor for this acquisition, but we followed a heterogeneous sample of patients, and the CCS may not be the most appropriate tool for use in studies with resistant pathogens [26]. The proportion of patients in study which became colonized each week increased and this is in accordance with the fact that patients who remain longer in hospital are exposed to more interventions (eg, by invasive procedures, contacts with healthcare workers, antimicrobials use) which may raise the risk of acquisition of a pathogen.

The number of colonized patients may have been underestimated since screening used only one site, and this is a limitation of the study. However, we wished to estimate how much work was involved in screening patients in a pragmatic study, in which the primary objective was to characterize the incidence of colonization by MRSA in an environment with high endemic levels. The lack of resources for a comprehensive strategy of universal MRSA screening demands a targeted approach that could result in a reduction of MRSA transmission, using the hospital capacity. Also, we did not adjusted for current use of prophylactic antibiotics used by oncologic patients (mainly, trimethropim-sulfamethozaxole), which may have affected colonization. Indeed, some patients received vancomycin because of suspected MRSA infection; this also could have biased the results of the nasal cultures, but the use of antimicrobial was not associated with colonization.

Almost 40% of the original sample that tested negative at admission was discharged at the weekend, without a second swab. This fact could have overestimated the incidence of colonization, because patients that had a shorter stay were less ill than the other patients, and have had a lower risk of becoming colonized.

Finally, if we consider the risk factors for adults at the time of admission, our findings suggest that the hospital should be able to screen 78% of those patients being admitted (all adult patients with admission in previous year and/or aged 60 years or more). This appears to be a high percentage if we consider isolating the patients while waiting the results of the cultures, as it would put them in isolation for 24% of their days in hospital. But if we look at the incidence rate found (5.5/1,000 patient-days), this measure could help lessen colonization pressure in the hospital. Another important point is that these newly-colonized patients would probably be readmitted, so maintaining the chain of MRSA transmission.

We also consider that this high prevalence of MRSA colonization at admission is a matter for concern, since the hospital emergency department is overcrowded and in this population the median time spent in the emergency department was two days before admission to an acute-care ward. Another Brazilian study has found that patients colonized or infected with multidrug-resistant bacterial organisms had a higher length of stay than controls in the ER, and a higher associated mortality [27]. The presence of colonized patients in a chronically overcrowded and understaffed ER may represent another substantial obstacle to implementing hospital infection control measures [28], especially where resources are limited, and thus requiring specifically customized strategies.

## Conclusions

Although MRSA infection is a very frequent pathogen in Brazilian hospitals, [2,3], there are few data regarding patient colonization [11-13]. Even though the universal screening is not a resolved issue, knowledge of the magnitude of colonization is invaluable for planning to control the pathogen. In a heterogeneous sample of clinical patients admitted to a Brazilian hospital, a high prevalence of MRSA colonization was found, and the results of the study provide the basis for better-targeted MRSA control policies aimed at specific groups of patients. The study also gives an estimate of the resources needed for a particular hospital to implement such interventions, especially where funds are limited.

## List of abbreviations

CCS: **Charlson Comorbidity Score**; ER: **Emergency room**; HAI: **Hospital-acquired Infections**; HIV: **Human immunodeficiency virus**; ICU: **Intensive Care Unit**; MRSA: **Methicillin-resistant *Staphylococcus aureus*; **PCR: **Polimerase chain reaction**; PR: **Prevalence ratio**; VRE: **Vancomicin-resistant enterococci**.

## Competing interests

The authors declare that they have no competing interests.

## Authors' contributions

HBS conceived the study, and participated in its design and coordination and helped to draft the manuscript. DPM and ALB participated in its design and carried out the microbiological analysis and detection of SCC*mecA*. SC participated in the statistical analysis and helped to draft the manuscript. RSK and MBW participated in the design of the study helped to draft the manuscript. All authors read and approved the final manuscript.

## Pre-publication history

The pre-publication history for this paper can be accessed here:

http://www.biomedcentral.com/1471-2334/10/328/prepub
